# Methodological issues of retrospective surveys for measuring mortality of highly clustered diseases: case study of the 2014–16 Ebola outbreak in Bo District, Sierra Leone

**DOI:** 10.1080/16549716.2024.2331291

**Published:** 2024-04-26

**Authors:** Grazia Caleo, Kamalini Lokuge, Katina Kardamanidis, Jane Greig, Jaroslava Belava, Emer Kilbride, Alhaji Sayui Turay, Gbessay Saffa, Ronald Kremer, Francesco Grandesso, Kostas Danis, Armand Sprecher, Gian Luca Di Tanna, Holly Baker, Helen A. Weiss

**Affiliations:** a Manson Unit, Médecins Sans Frontières (MSF), London, UK; b MRC International Statistics and Epidemiology Group, Faculty of Epidemiology and Population Health, London School of Hygiene and Tropical Medicine, London, UK; c National Centre for Epidemiology and Population Health, Research School of Population Health, Australian National University, Canberra, Australia; d Public Health Department MSF, Amsterdam, The Netherlands; e Vancouver Coastal Health, Vancouver, BC, Canada; f District Health Management Team, Ministry of Health and Sanitation, Bo, Sierra Leone; g Department of Epidemiology and Training, Epicentre, Paris, France; h Santé publique France, The French National Public Health Agency (SpFrance), Saint-Maurice, France; i Medical Department, Médecins sans Frontières, Brussels, Belgium; j The George Institute for Global Health, University of New South Wales, Sydney, Australia

**Keywords:** Ebola virus disease, design effects, cluster surveys, mortality, highly-clustered data

## Abstract

**Background:**

There is a lack of empirical data on design effects (DEFF) for mortality rate for highly clustered data such as with Ebola virus disease (EVD), along with a lack of documentation of methodological limitations and operational utility of mortality estimated from cluster-sampled studies when the DEFF is high.

**Objectives:**

The objectives of this paper are to report EVD mortality rate and DEFF estimates, and discuss the methodological limitations of cluster surveys when data are highly clustered such as during an EVD outbreak.

**Methods:**

We analysed the outputs of two independent population-based surveys conducted at the end of the 2014–2016 EVD outbreak in Bo District, Sierra Leone, in urban and rural areas. In each area, 35 clusters of 14 households were selected with probability proportional to population size. We collected information on morbidity, mortality and changes in household composition during the recall period (May 2014 to April 2015). Rates were calculated for all-cause, all-age, under-5 and EVD-specific mortality, respectively, by areas and overall. Crude and adjusted mortality rates were estimated using Poisson regression, accounting for the surveys sample weights and the clustered design.

**Results:**

Overall 980 households and 6,522 individuals participated in both surveys. A total of 64 deaths were reported, of which 20 were attributed to EVD. The crude and EVD-specific mortality rates were 0.35/10,000 person-days (95%CI: 0.23–0.52) and 0.12/10,000 person-days (95%CI: 0.05–0.32), respectively. The DEFF for EVD mortality was 5.53, and for non-EVD mortality, it was 1.53. DEFF for EVD-specific mortality was 6.18 in the rural area and 0.58 in the urban area. DEFF for non-EVD-specific mortality was 1.87 in the rural area and 0.44 in the urban area.

**Conclusion:**

Our findings demonstrate a high degree of clustering; this contributed to imprecise mortality estimates, which have limited utility when assessing the impact of disease. We provide DEFF estimates that can inform future cluster surveys and discuss design improvements to mitigate the limitations of surveys for highly clustered data.

## Paper Context

- **Main findings:** For humanitarian organizations it is imperative to document the methodological limitations of cluster surveys and discuss the utility.
- **Added knowledge:** This paper adds new knowledge on cluster surveys for highly clustered data such us in Ebola virus disease.
- **Global health impact of policy and action:** We provided empirical estimates and discuss design improvements to inform future study.

## Background

In humanitarian contexts, surveillance is the accepted gold standard to measure public health outcomes and to estimate the impact of a crisis (including mortality) [1]. During the 2014–2016 Ebola virus diseases (EVD) outbreak in Sierra Leone, vital registration and surveillance systems were weak [2], necessitating reliance on estimates of the direct and indirect impacts of the outbreak based on mathematical modelling and retrospective analysis of burial and health facility data [3–6].

Population-based surveys can supplement surveillance data to estimate the severity of a crisis for a range of purposes, including operational planning (e.g. to prioritise areas for intervention), and advocacy [7,8]. A previous study explored the validity of cluster surveys versus systematic sampling methods for measuring crude mortality [9]. The authors reported that both designs yielded similar estimations, but warned that their findings may not be generalisable and advocated further research to address key methodological limitations of cluster surveys in humanitarian contexts. These include failure to calculate the optimal sample size, to sample proportionate to population size (PPS), to weight the sample during analysis, and to consider the design effects (DEFF) when calculating precision [10]. DEFF considers that individuals (or households) living close to each other are more similar than individuals (or households) living far away at a random distance. In cluster surveys, this may lead to high intra-cluster correlation, high DEFF, reduction in the effective sample size and therefore loss in precision of the estimates. When designing a cluster survey, the value of the expected DEFF is multiplied to the sample size to compensate for the loss of precision [11]. This is particularly important when measuring EVD burden, compared to other human-to-human transmitted infectious diseases (e.g. measles), since Ebola virus requires close physical contact for infection, leading to violation of the assumption of data independency. While this topic has been explored in veterinary epidemiology [12] where animals are frequently clustered at the herd/farm level, there is, however, a paucity of literature for human studies [13]. Thus, it is recommended to publish empirical estimates of design effects DEFFs to inform future studies.

This paper assesses the utility, and limitations of cluster surveys for highly clustered data, using data from two cluster surveys carried out in rural and urban areas of Bo District, Sierra Leone, during the 2014–2016 EVD outbreak. The surveys were designed to estimate mortality (due to EVD and non-EVD) and morbidity in areas where, due to the EVD outbreak, Médecins Sans Frontières (MSF) suspended critical health interventions and refocused on EVD care.

We planned the surveys knowing that EVD case clustering might affect survey estimates, therefore at design and implementation attempts were taken to account for the highly-clustered distribution of disease outcomes.

The aims of this paper are to i) report crude and adjusted EVD mortality rate estimates, ii) provide the first reported estimated DEFFs for EVD and non-EVD mortality during an EVD outbreak; iii) discuss the methodological limitations and the operational utility of estimated mortality rates from cluster-sampled survey when the DEFF is high.

## Methods

### Study setting and population

In September 2014, MSF opened a 100-bed Ebola Management Center (EMC) in Bo township to reduce EVD mortality and transmission in the area. Alongside EMC activities, MSF conducted EVD outreach activities in Bo district, focusing on social mobilisation, support of survivors, case finding and case investigation efforts conducted in collaboration with the District Ebola Response Committee (DERC).

Prior to the EVD outbreak, MSF was supporting the running of a 200-bed secondary-level referral hospital in Gondama, just outside Bo town. The hospital was considered a lifeline for children and pregnant women coming from Bo and other districts, providing more than 8,000 paediatric and 2,500 emergency obstetrical and gynaecological admissions per year [14]. However, in October 2014, MSF was forced to suspend health services due to the increasing risk of EVD nosocomial transmission and concerns about staff safety [15].

The closure of the Gondama referral hospital was perceived to have contributed to an increase in mortality in the area. Thus, in the absence of strong routine surveillance and vital statistics, we conducted two surveys in MSF catchment areas to estimate EVD and non-EVD mortality and morbidity in Bo district during the Ebola outbreak. One survey was carried out in Bo rural areas which consist of 15 chiefdoms and 969 villages, with an estimated population of 538,751. The second survey was carried out in Bo town (urban area of Bo District) consisting of 20 sections, with an estimated population of 178,446. Further rationale to carry out two independent surveys was based on anticipated differences in terms of clustering between urban and rural areas.

### Study design

We used a two-stage population-based cluster survey design, an established methodology to estimate mortality rates in humanitarian and crisis settings [16–18].

### Recall period

In Sierra Leone, the first EVD cases were reported on 24 May 2014 [19]. The last confirmed case in Bo District was discharged on 26 January 2015 (the last confirmed case in Sierra Leone was reported in March 2016). The recall period started from 24 May 2014 for both surveys and ended on 1^st^ of April 2015 for the rural survey (313 days), and the 9^th^ of April 2015 for the urban survey (321 days), which corresponded to the start of the survey in each location.

Group discussions with the Sierra Leone study team were used to design a local events calendar of the recall period to help interviewees to report when a death occurred in their household. The calendar incorporates relevant national awareness days, religious observance events and community events, as well as salient events linked to the EVD outbreak (e.g. the declaration of a state of emergency) [8,20]. Study teams recorded any changes in household composition (births, deaths, and in- and out-migration) and illnesses during the established recall period.

### Sample size

As with other infectious diseases transmitted by human to human contacts (e.g. due to attending the same funerals and/or caring for a sick relative), there is substantial geographical heterogeneity of EVD infection, with people living in an Ebola-affected household or village having a high household and village-level risk of EVD infection and death [21].

Prior to the EVD outbreak, the all-cause mortality rate in Sierra Leone was estimated between 0.5 and 0.7 deaths per 10,000 people per day [22]. In the absence of published DEFF estimation from prior EVD cluster surveys, we considered a range of sample size scenarios when designing our surveys (see Table A1), using different estimates of expected crude mortality rate (CMR), required precisions and assumed design effects [22,23]. Based on these simulations, the most likely estimate was considered to be a CMR of 1.0 deaths/10,000 person-days with a precision of ±0.5 deaths and a design effect of 4. The DEFF value of 4 was considered at that time to be the worst scenario in terms of clustering in either area. Using ENA (2011) software, the required sample size for each area was calculated as 2390 individuals in 483 households. Since the value of DEFF increases with cluster size and reduces with cluster number, in the attempt to reduce the DEFF, we opted to increase the minimum recommended number from 30 to 35 clusters of 14 households per area for a total of 70-clusters [24]. The total number of clusters was considered sufficiently large in the light of the expected DEFF along with human and logistic constraints of implementing operational research under outbreak conditions.

### Sampling

Population estimates in Bo district (by village) and Bo town (by section) were obtained from the Local Ministry of Health and Sanitation (MOHS). These lists constituted the sampling frame from which the clusters were selected. In the first stage, villages/sections were selected with PPS. In the second stage, the starting household was chosen within a village (rural area) or within a section (urban area) using a variation of the standard World Health Organization (WHO) Expanded Programme on Immunization (EPI) methodology [25]. After having identified the geographical centroid of the village/sector with the help of the head of the village/sector, a random direction was identified by spinning a pen. Study teams then walked in a straight line to the edge of the village/sector and assigned a progressive number to each household in that line. A random number between 1 and the number of households counted was drawn using a random number table. The households tagged with the drawn number was the first household to be surveyed.

In a bid to further reduce the DEFF that might occur by including geographically close households, a step between household was added to identify subsequential households in the cluster. We selected every n^th^ household, where n was the total estimated households in the village/section divided by the number of households to be included. Subsequent (n^th^) households were selected by counting households to the left. If a household was empty, two further attempts were made later the same day before replacement. Replacement, including due to refusal, was with the next closest household to the left.

### Definitions

The WHO EVD case definitions were used to define suspect cases (Box 1) [26]. In addition, EVD suspected and probable cases were further asserted by triangulating of the following information: (1) meeting the WHO criteria of suspected/probable case (2), history of household being under quarantine (3), history of household being under contact tracing (4), history of being referred to the MSF Bo EMC. Classification of causes of death or major morbidity are reported in Table A2. A household was defined as a person or a group of persons, related or unrelated, who lived together and who shared a common source of food. The head of the household was defined as a person aged 18 years and older who could give information on demographics, illness, and mortality in his/her household and was present in the household during the recall period.

**Box 1:** World Health Organization (WHO) EVD case definitions were used to define suspect, probable, and confirmed cases

Suspect	Probable	Laboratory confirmed
a. Any person, alive or dead, suffering or having suffered from a sudden onset of high fever and having had contact with: - a suspected, probable or confirmed Ebola or Marburg case; - a dead or sick animal; OR b. Any person with sudden onset of high fever and at least three of the following symptoms: - headaches – lethargy – anorexia/loss of appetite – aching muscles or joints – stomach pain – difficulty swallowing – vomiting – difficulty breathing – diarrhoea – hiccups; OR c. Any person with inexplicable bleeding; OR d. Any sudden, inexplicable death	a. Any suspected case evaluated by a clinician; OR b. Any deceased suspected case (where it has not been possible to collect specimens for laboratory confirmation) having an epidemiological link with a confirmed case	a. Any suspected or probable cases with a positive laboratory result. Laboratory confirmed cases must test positive for the virus antigen, either by detection of virus RNA by reverse transcriptase-polymerase chain reaction (RT- PCR), or by detection of IgM antibodies directed against Marburg or Ebola.

Quarantine was defined as a household reporting separation from the rest of the community (i.e. the household was cordoned off) and restriction of movement by the local authority following a positive EVD result in the household. Contact tracing was defined as a process of identifying, listing, and monitoring persons who had direct exposure (physical contact between infected person and susceptible person) or indirect exposure (e.g. contaminated surfaces or objects) to any confirmed EVD case within the past 21 days.

### Data management

Interviews were conducted with the head of each selected household. A trained MSF study team elicited information on household members, births, arrivals, departures, illnesses, deaths, place and circumstance of death. Medical records at the household level were used to re-build the possible cause and time of illness and death, if available. Data on whether and when the household had been placed under quarantine were collected. Additionally, information on whether any household members were placed under contact tracing was also gathered. The questionnaire and consent forms were verbally translated into the dominant local language Mende, which does not have a written tradition, and back-translated into English to ensure consistency. The study team were bi-lingual (speaking English and Mende). Group consensus on translations was sought during the training. During data collection, the research teams were composed of two surveyors who supported each other on translation consistency. Questionnaires were piloted prior to beginning the study. The detailed study protocol is publicly available on the MSF research platform (https://remit.oca.msf.org/studies/159).

### Data analysis

We present a descriptive analysis of the mean or median (range) of numerical variables and proportions with 95% confidence intervals (CI) for categorical variables. Mortality rates per 10,000 per day were estimated using the mid-point population estimates as the denominator. Mid-point populations accounted for changes in household composition (births, deaths, and in- and out-migration) during the recall period. Rates were estimated for all-cause, all-age, under-5 mortality and EVD-specific mortality. Stratified linear and logistic regression models for continuous and binary outcome variables, respectively, were fitted adjusting for a-priori defined variables of age and sex. We estimated the crude and adjusted mortality rates, and incidence rate ratios (IRR) using Poisson regression. All analyses were conducted separately by area and accounted for the survey sampling weights and the DEFF in each area. Data cleaning and statistical analysis were conducted using STATA v15 (Stata Corporation, TX, USA).

### Ethical approval

The study protocol was approved by the Ethics Review Board of MSF, the Internal Review Board of the Sierra Leone MoHS, and the London School of Hygiene & Tropical Medicine (LSHTM). Approval to conduct the study was obtained from traditional authorities in all study sites prior to data collection. Participation was voluntary. Verbal informed consent for participation was obtained from the head of each household after a briefing about the aim of the study, the questions, survey and how their answers would be recorded, stored and used, duration of the questionnaire, and the option to end the interview or withdraw from the research at any time if wished. Confidentiality was protected during data collection and analysis. No personal identifying information was collected.

## Results

### Overview

The surveys were conducted in 70 clusters (35 clusters in each rural and urban areas) of 14 households (total 980 households; 6,522 individuals). Four households refused to participate (one in the rural area and three in the urban area) and were replaced by the next consenting household. The rural area had a higher proportion of children under 5 years (14.4% 95%CI: 13.3–15.5%) than the urban area (9.4%, 95%CI: 8.4–10.4%) (*p* < 0.001). The proportion of women was lower in the rural area (51.4%, 95%CI: 49.6–53.2%) than in the urban area (54.6%, 95%CI: 52.9–56.3%) (*p* = 0.01) (Table 1).

**Table 1.** Households characteristics and movements according to area, mortality studies Bo District.

Variable	Urban	Rural	p-value for difference
No. clusters	35	35	
No. households sampled	490	490	
No. individuals at baseline (start recall period)	3,266	3,048	
No. departure (deaths/out-migration)	122	140	
No. arrivals (births/in-migration)	108	100	
Mid-point population	3,259	3,028	
No.Children <5 years	316	452	
No.Female	1844	1619	
Total number of individuals in study	3,374	3,148	
Mean household size (95%CI)	8.6	7.9	0.15
	(7.9–9.2)	(7.3–8.6)	
Mean age (95%CI)	22.9	22.7	0.71
	(22.3–23.5)	(21.9–23.5)	
% Children <5 years (95%CI)	9.4	14.4	<0.001
	(8.4–10.4)	(13.3–15.5)	
% Female (95%CI)	54.6	51.4	0.01
	(52.9–56.3)	(49.6–53.2)	

### Morbidity

Overall, 9.0% (*n* = 586, 95%CI: 7.2–10.1%) of the population surveyed reported that someone had been sick at least once during the recall period. Prevalence of any morbidity was reported more frequently among children under 5 (240/768; 29.2%, 95% CI: 22.8–36.6) compared to over 5 years old (346/5754; 5.4%, 95% CI: 4.4–6.7%) (*p* < 0.001). The most frequently reported illness (all ages combined) was malaria/fever (*n* = 358, 61.1%). Prevalence of suspected/probable EVD was 4.9% (*n* = 29 of 586).

### EVD survivors

In total, nine people reported being EVD survivors, all of whom reported being admitted to MSF Bo EMC. Of these, seven reported that their household was put under quarantine and contact tracing following their positive test. Six of the nine cases reported signs, symptoms and/or contact history compatible with the suspect/probable WHO EVD case definition.

### Mortality

Overall, 36/70 clusters (51.4%) reported deaths (16 clusters in the rural and 20 in the urban area). In total, 64 deaths were reported, of whom 18 were among children aged under 5 years old, giving crude and under-five mortality rates of 0.35/10,000 person-days (95%CI: 0.23–0.52) and 0.91/10,000 person-days (95%CI: 0.54–1.51), respectively (Table 2). All-cause mortality in the rural area was higher than in the urban area (adjusted IRR 1.25; 95% CI: 0.67–2.33), but this difference was not statistically significant (Table 2).

**Table 2.** Reported deaths and crude and under-5-year mortality rates, crude and adjusted incidence rate ratios, mortality studies Bo District.

	N of deaths		Mortality rate (all ages) per 10,000-person-days			Under-five mortality rate per 10,000-person-days		
Area	Total	<5 years	Rate	[95% CI]	Design Effect	Rate	[95% CI]	Design Effect
Overall	64	18	0.35	[0.23–0.52]	2.86	0.91	[0.54–1.51]	1.43
Urban	28	4	0.27	[0.19–0.38]	0.45	0.41	[0.13–1.30]	0.60
Rural	36	14	0.38	[0.23–0.62]	3.44	1.03	[0.59–1.77]	1.52
Crude incidence rate ratio (IRR)			1.41	[0.77–2.60]		2.51	[0.69–9.14]	
Adjusted IRR^a^			1.25	[0.67–2.33]		2.56	[0.71–9.28]	

^a^Adjusted for age group and sex.

The most frequently reported causes of death were EVD (31.2%, *n* = 20) and malaria/fever (18.7%, *n* = 12). EVD was the main reported cause of death among individuals aged over 5 years (39.1%, *n* = 18), while malaria/fever was the main cause among children under-5s (50.0%, *n* = 9) (Table 3). For two children aged under 5 the cause of death was attributed by family members to EVD; both were in rural areas and in households experiencing more than one EVD case.

**Table 3.** Reported causes of death by age group, mortality studies Bo District.

Cause of death	Five years old and over (*N* = 46)		Under five years old (*N* = 18)	
	N	%	N	%
EVD	18	39.1	2	11.1
Chronic diseases	8	17.4	-	-
Other*	6	13.0	1	5.5
Old Age	5	10.7	-	-
Malaria/Fever	3	6.5	9	50.0
Unknown	3	6.5	-	-
Trauma/Accident	2	4.3	1	5.5
Convulsion	-	-	2	11.1
Death during pregnancy or childbirth	1	2.2	-	-
Neonatal death	-	-	3	16.7

*Sickle Cell Disease, Candidiasis, ‘Witch Gun’, Swollen Foot, Abdominal Pain, Herpes Zoster, Hernia.

All households where EVD deaths were reported experienced quarantine and all except one experienced contact tracing. Nine of the 20 EVD deaths (45.0%) met the suspect/probable WHO EVD case definitions. The place of death was at home (*n* = 9, 45.0%), at Bo MSF EMC (*n* = 6, 30.0%), at a non-Ebola health facility (*n* = 4, 20.0%) or in an ambulance (*n* = 1, 5.0%).

The overall EVD-specific mortality rate was 0.12/10,000 person-days (95% CI: 0.05–0.32). EVD-related mortality was higher in rural area compared with urban (adjusted IRR 2.61; 95% CI: 0.65–10.35); confidence intervals for EVD-specific mortality rates were wide so despite different point estimates, it was not possible to conclude that they were significantly different (Table 4).

**Table 4.** Reported deaths, EVD specific and non-EVD specific mortality rates, crude and adjusted incidence rate ratio, mortality studies Bo District.

	N of deaths		EVD specific mortality rate per 10,000-person-days			Non-EVD-specific mortality rate per 10,000-person-days		
Area	EVD	Non-EVD	Rate	[95% CI]	Design Effect	Rate	[95% CI]	Design Effect
Overall	20	44	0.12	[0.05–0.32]	5.53	0.22	[0.16–0.32]	1.53
Urban	6	22	0.06	[0.02–0.14]	0.58	0.21	[0.14–0.31]	0.44
Rural	14	22	0.15	[0.05–0.43]	6.18	0.23	[0.15–0.37]	1.87
Crude IRR			2.56	[0.64–10.26]		1.10	[0.59–2.04]	
Adjusted IRR^a^			2.61	[0.65–10.35]		0.91	[0.50–1.65]	

^a^Adjusted for age group and sex.

### Clustered events and design effects

The 29 EVD cases were reported in 20 households in 12 clusters, while the 358 malaria/fever cases were reported in 272 households in 62 clusters. The DEFF for EVD infection was 28% higher compared to malaria/fever infection (7.01 vs 5.47) (Table 5).

**Table 5.** Reported malaria and EVD infections, morbidity rates and design effect, mortality studies Bo District.

Area	N of cases		Malaria/fever morbidity rate (*)			Ebola morbidity rate (*)		
	Malaria/fever	Ebola	Rate	[95% CI]	Design Effect	Rate	[95% CI]	Design Effect
Overall	358	29	1.74	[1.36–2.22]	5.47	0.17	[0.07–0.42]	7.01
Rural	163	18	1.72	[1.25–2.35]	6.72	0.19	[0.06–0.55]	8.21
Urban	195	11	1.86	[1.39–2.48]	2.07	0.11	[0.05–0.21]	0.62

(*) Rate per 10,000 persons per Day.

The 20 EVD deaths were reported in 15 households in 9 clusters, with the 44 non-EVD deaths reported in 40 households in 31 clusters. The DEFF for EVD-specific mortality rate was 3.6 times higher compared to non-EVD mortality rate (5.53 vs 1.53) (Table 4).

Five of six clusters (83.3%) reporting more than one EVD case were in the rural area. Death at home was more frequent among clusters reporting more than one EVD case compared to clusters reporting one EVD case, however the difference was not statistically significant (26.7% vs 7.1%, *p* = 0.39).

Eighteen EVD cases occurred in 4 of 35 rural clusters (11%) compared to 11 EVD cases reported in 8 of 35 urban clusters (23%). Two rural clusters reported six and seven cases, respectively. The DEFF for EVD-specific morbidity (overall 7.01) was 13 times higher in the rural area compared with the urban area (8.21 vs 0.62) (Table 5). The DEFF for EVD-specific mortality (overall 5.53) was 11 times higher in the rural area compared with the urban area (6.18 vs 0.58) (Table 4).

## Discussion and recommendations

To our knowledge, this is the first and largest population-based study conducted during the 2014–2016 EVD outbreak in Sierra Leone, and the first study to estimate mortality rates and DEFF separately for urban and rural areas and for EVD and non-EVD, respectively. Estimated CMRs were lower than expected, despite the closure of the Gondama referral hospital and the disruption of the health care system caused by the EVD outbreak.

In our sample, a small proportion of households and clusters reported EVD deaths and cases. Households in the rural area experienced a higher number of EVD deaths at home, and more clusters with more than one EVD case, possibly due to limited access to care along with weaker surveillance system delaying reaching rural areas [27]. EVD community death is one of the key WHO indicators to evaluate the performance of and readiness EVD response [28]. It could be further used by public health actors to prioritise those households and communities where secondary transmission is more likely to occur for interventions such as EVD vaccination, and more rigorous contact tracing to ensure timely access to care, in particular when transmission is high and human resources to address the outbreak are scarce. Furthermore, this indicator could be combined with anthropological assessments (e.g. on community perceptions and experience, healthcare-seeking behaviour, health services utilisation) to adapt interventions and improve collaboration with affected communities.

Malaria/fever was the most common illnesses reported in all ages, and the main cause of death for under-5 year olds. Surveillance, at the time, was focused on EVD detection with low attention to provide care for other endemic diseases like malaria [2]. As previously suggested, malaria interventions should be prioritised in EVD outbreak responses, in particular for children aged under-5 and in rural settings [29]. This will mitigate the additional morbidity and mortality burden, and stimulate the community to report to surveillance if health interventions are consistent with population needs.

As with other EVD studies, the under-reporting of deaths due to the fear stigma from other households and dissatisfaction with how deaths are being handled (e.g. safe burial teams not accounting for culturally important burial practices) cannot be excluded [30]. In addition, we cannot exclude that the national policy of mandatory reporting of death might have discouraged households to report deaths [31].

However, a mitigating factor is that MSF was not associated with the enforcement of punitive measures (e.g. fine for sick people if concealed, and/or violation of quarantine [32]). MSF had an established presence in Bo preceding the Ebola outbreak as a provider of free healthcare, which further supported trust with the community. Further, there was a high response rate to the survey; even those who were not randomly selected expressed their willingness to participate (but were not included in the study), suggesting that the survey was well perceived by the community, and therefore desirability bias could not be excluded.

Only 45% of the reported EVD deaths met the WHO case definition, thus misclassification of the cause of deaths cannot be excluded, with possible over/under estimation of EVD mortality. Nevertheless, the poor performance of WHO EVD case definition is consistent with a previous study that showed that the WHO EVD case definition has a specificity of 36% (thus 64% of people identified as EVD suspected have potentially other diseases) and a sensitivity of 81% (thus 19% of patients with EVD do not meet the case definition and would be otherwise missed) [33].

Furthermore, potential misclassification of the timing of death may have occurred, but, when available, we used medical records, clinical/contact history, information on quarantine and contact tracing, and a calendar of salient events to mitigate misclassification.

Two other surveys, in Freetown (Sierra Leone) and Monrovia (Liberia), estimated CMR and EVD-specific mortality rates covering a recall period which overlapped with our study [34,35]. Both were conducted with the assumption of an increase in mortality due to the outbreak. The Freetown study used our methodology (i.e. a two-stage population-based cluster but with a lower predicted DEFF of 1.5) [34]. In Monrovia, a simple random sample of telephone numbers with remote interview was implemented, since a two-stage population-based cluster was deemed risky for the study team due to EVD transmission [35]. In Freetown, the CMR was 0.52/10,000 persons/day (95%CI: 0.29–0.76) and Ebola-specific mortality rate 0.19/10,000 person-days (95% CI: 0.01–0.38) [34]. DEFF was not reported in this study. In Monrovia, the CMR was 0.33/10,000 person-days (95%CI: 0.25–0.43) and Ebola-specific mortality rate 0.06/10,000 person-days (95%CI: 0.03–0.11) [35]. Both author groups did not discuss whether clustering of Ebola cases might have affected their estimates, and both attributed the low mortality rates to improved access to care, enhanced hygiene practices due to the outbreak and low reporting of deaths in particular for under-5s. These factors may also have contributed to the lower than expected mortality seen in our study.

### Methodological reflections and design improvements

The elevated clustering of EVD cases resulted in higher-than-expected DEFF, holding imprecise mortality estimates, which had limited utility when assessing the impact of EVD disease and eventually made interpretation more difficult. The elevated DEFF in the rural area was mainly due to the high proportion of EVD deaths in one cluster, and the close geographical distribution of cases within clusters leading to imprecise estimation. This problem was observed in another previous study on EVD [21]. This is a key limitation of the methodology used that could be addressed by adopting the methods we use or seeking alternative approaches.

In our study, in an attempt to mitigate the impact of clustering and therefore the DEFF, we adapted the within cluster sampling strategy, whereby rather than selecting geographically clustered households, every n^th^ household was selected (where n was the total estimated household in the village/section divided by the number of households to be included). This was a pragmatic approach that was not sufficient to address the issue. More sophisticated procedures, such as spatial sampling, should be considered to improve randomness within the cluster [36]. Apart from using spatial sampling in retrospect, we should have considered increasing the sample size or, with the same sample size increase, increasing the number of clusters and consequently reducing their size in a bid to obtain a low DEFF [24] and/or consider using the variance partition coefficient to calculate separate sample sizes according to groups that show significantly different heterogeneities [37]. This latter approach has been used in veterinarian epidemiology to analyse herd-level predictors, which measures the clustering of infection/disease for individuals with a common risk profile (e.g. animals in the same herd). However, all efforts to obtain better precision have to be balanced with resources and field capacity that can be mobilised.

Alternative methods could be a capture-recapture approach [38], which estimates the size or prevalence by exploring the overlaps and events not captured by two or more independent lists, as suggested by another study conducted in Monrovia during the early phase of the EVD outbreak [39]. In this study, the capture-recapture approach showed that the likely number of EVD cases was at least three times higher than the number officially reported [39]. Lack of access to at least two independent EVD case lists hampered the ability to consider a capture-recapture and to assess the robustness of our survey estimates.

Another approach coud be a purposeful selection of clusters guided by knowledge of the spatial distribution of the outcome, instead of using the village sampling frame [40]. This method allows the investigation of clusters with reported transmission, thus potentially providing more robust estimates and better allocations of resources. However, this has the limitation of giving estimates only relevant for the purposively selected areas (with limited application to the wider population) or exclude areas affected but not identified as such, or select area due to other factors (e.g. lack of access, or unwillingness to report).

Other authors have used a snowball approach to estimate maternal deaths [41], a method that recruits subjects who meet the characteristics of interest who then refer to other people with the same characteristics, and so on, creating a ‘snowball rolling down’ effect [42]; this approach has proven to be cost-effective to capture visceral leishmaniasis deaths [43]. More recently, a snowball approach was proposed to estimate the impact of SARS-CoV-2 transmissibility starting from contact networks [44]. Despite the non-stochastic sampling procedure, this approach may be useful in some specific circumstances, for example, to replace exhaustive surveys in small areas with accurate population estimates. A similar approach, called the informant method, that gathers information on health events from knowledgeable individuals in the community such as key informants’ healthcare provides, have been evaluated by other authors to estimate real-time mortality [45]. However, this approach was evaluated for short recall periods (e.g. 30–60 days) and held results with sensitivity below 80% when compared to the capture-recapture method.

Finally, integrating qualitative methodologies into existing quantitative study approaches would provide additional crucial information (e.g. implementation of control measures, population compliance, health-seeking behaviour, barrier to access to care) to support interpretation of estimates. For instance, in retrospect, we could have included in-depth interviews with burial teams and households who report deaths to complement and corroborate estimates.

In Table 6, we summarise challenges, proposed methods or design improvements to mitigate the limitations of surveys for highly clustered data, along with requisites and additional considerations.

**Table 6.** Challenges, proposed methods and design improvements and considerations.

Challenges observed	Proposed alternative methods and/or improvements of two-stage cluster survey	Requisites & considerations
Selection of a large number of unaffected clusters and households resulting in inaccurate estimates of EVD outbreak burden/impact	Consider to use surveillance list or a purposeful selection of clusters as a sampling frame instead of the list of villages.	Access to surveillance data.Previous knowledge of geographical distribution of health characteristics under investigation.Potential limited representativeness of the results (i.e, estimates only relevant for the purposively selected areas).
Differences in the geographical distribution across areas that might affect DEFF andprecision of estimates	Consider different selection procedures in the second sampling stage (e.g. spacial sampling)	Access to updated satellite images and field validation of households distributions
	Increase the number of clusters to increase geographical variability	Large logistical and human resources
	Consider sampling methods used in veterinary epidemiology that use the variance partition coefficient (VPC), which measures the clustering of infection/disease for individuals with a common risk profile (e.g. animals in the same herd).	Knowledge of social and epidemiological factors (e.g. review of chain of transmission, social factors/traditions/coping meccanisms that could influence patterns of transmission, control measures in place, access to care)Sample size estimates are obtained separately for those groups that exhibit significantly different heterogeneity.
	As above plus consider additional variables for stratification influencing DEFF: age (e.g. under 5 years olds vs 5 and over) and other social/epidemiological parameters influencing transmission.	As above
Identification of EVD-affected households	Involve community chiefs, community health workers, and/or key local stakeholders to support identification of affected EVD households.Consider non-probabilistic methods (e.g. snowball approach) to identify additional affected household and use whole population as denominator.Consider informant method approaches to count deaths occurring in a short recall period.	To be used only to replace exhaustive surveys.Breach in confidentiality and possible increase of stigma for affected households.Short recall period, accurate population estimation, along with an heterogeneous group of key informants.
Robustness of estimations	Consider to designing a capture-recapture study using different sources (e.g. burial data, EVD vaccination list, local key stakeholders).	Access and/or creation of independent lists.
Interpretation of estimates	Mixed-method approaches using qualitative and quantitative data.	Require additional qualitative research skills and resources.Disclosure of information around circumstance of death could be challenged, in particular for the qualitative component.

## Conclusion

For humanitarian organisations, it is imperative to document the methodological limitations of studies and discuss the utility of estimates generated by common epidemiological tools used to quantify burdens and needs, in order to ensure accountability with affected populations and effective use of resources in public health emergencies.

Our findings demonstrate a high degree of clustering in current methodologies for community-based surveys of EVD. The empirical DEFF estimates we provide can inform more robust study designs in future retrospective surveys of highly clustered diseases such as EVD. If vital registration and/or comprehensive routine surveillance is not feasible, alternative survey designs including mixed-method studies and increasing number of clusters would improve the utility of information collected.

## Data Availability

Data are available under the MSF data sharing policy. Requests to access data can be made to data.sharing@msf.org.

## Appendix Appendix

**Table A1.** Sample size estimation scenarios*.

Scenario	Estimated CMR (per 10,000/day)	Desired precision of CMR	Design effect**	Total population required	# HH	If 30 clusters, # HH per cluster	If 35 clusters, # HH per cluster
1	0.5	0.5	4	1195	241	8.0	6.9
2	0.5	0.5	2	597	121	4.0	3.5
3	0.5	0.25	**4**	4779	966	32.2	27.6
4	0.5	0.25	2	2390	483	16.1	13.8
5	0.7	0.5	4	1673	338	11.3	9.7
6	0.7	0.5	2	836	169	5.6	4.8
7	0.7	0.25	4	6691	1352	45.1	38.6
8	0.7	0.25	3	5018	1014	33.8	29.0
9	0.7	0.25	2	3346	676	22.5	19.3
10	**1**	**0.5**	**4**	**2390**	**483**	**16.1**	**13.8**
11	1	0.5	2	1195	241	8.0	6.9
12	1	0.25	4	9559	1931	64.4	55.2
13	1	0.25	2	4779	966	32.2	27.6

*Constants: Recall period 280 days; household size 5.5; non-response rate 10%.

**Influenced by number of clusters selected.

**Table A2.** Classification of reported causes of deaths or major morbidity reported.

Causes of deaths	Main reported symptoms and sign	Recall period
Malaria	Fever with or without other symptoms e.g. Headache Fatigue	From 24 May 2014 to 1 April 2015 in the rural area (313 days) From 24 May 2014 to 9 April 2015 in the urbal area (321 days)
Convulsion	Convulsion	
Chronic diseases	Hypertension Stroke Breast cancer	
Old Age	Age between 70 to 99 without any other reported symptoms and sign	
Trauma/Accident	Drown Car accident	
Death during pregnancy or childbirth	Maternal deaths from any cause related to pregnancy during pregnancy and childbirth or within 42 days of termination of pregnancy, irrespective of the duration and site of the pregnancy.	
Neonatal death	Death of a live born infant, regardless of gestational age at birth, within the first 28 completed days of life	
EVD	See Box 1	

## References

[1] Checchi F, Warsame A, Treacy-Wong V, Polonsky J, van Ommeren M, Prudhon C. Public health information in crisis-affected populations: a review of methods and their use for advocacy and action. Lancet. 2017;390:2297–12. doi: 10.1016/S0140-6736(17)30702-X 28602558

[2] Njuguna C, Jambai A, Chimbaru A, Nordstrom A, Conteh R, Latt A, et al. Revitalization of integrated disease surveillance and response in Sierra Leone post Ebola virus disease outbreak. BMC Public Health. 2019;19:364. doi: 10.1186/s12889-019-6636-1 30940125 PMC6444503

[3] Parpia AS, Ndeffo-Mbah ML, Wenzel NS, Galvani AP. Effects of response to 2014-2015 Ebola outbreak on deaths from malaria, HIV/AIDS, and tuberculosis, West Africa. Emerg Infect Dis. 2016;22:433–441. doi: 10.3201/eid2203.150977 26886846 PMC4766886

[4] Sochas L, Channon AA, Nam S. Counting indirect crisis-related deaths in the context of a low-resilience health system: the case of maternal and neonatal health during the Ebola epidemic in Sierra Leone. Health Policy Plan. 2017;32:iii32–iii9. doi: 10.1093/heapol/czx108 29149310

[5] Oduyebo T, Bennett SD, Nallo AS, Jamieson DJ, Ellington S, Souza K, et al. Stillbirths and neonatal deaths surveillance during the 2014-2015 Ebola virus disease outbreak in Sierra Leone. Int J Gynaecol Obstet. 2019;144:225–231. doi: 10.1002/ijgo.12722 30467853 PMC6540087

[6] Bolkan HA, Bash-Taqi DA, Samai M, Gerdin M, von Schreeb J. Ebola and indirect effects on health service function in Sierra Leone. PLoS Curr. 2014;6. doi: 10.1371/currents.outbreaks.0307d588df619f9c9447f8ead5b72b2d PMC4318968 25685617

[7] Checchi F, Roberts L. Documenting mortality in crises: what keeps us from doing better. PLoS Med. 2008;5:e146. doi: 10.1371/journal.pmed.0050146 18597552 PMC2443202

[8] Checchi F. Estimation of population mortality in crisis-affected populations: guidance for humanitarian coordination mechanisms. 2018. Available from: https://www.who.int/health-cluster/resources/publications/LSHTM-Mortality-Estimation-Options-oct2018.pdf

[9] Rose AM, Grais RF, Coulombier D, Ritter H. A comparison of cluster and systematic sampling methods for measuring crude mortality. Bull World Health Organ. 2006;84:290–296. doi: 10.2471/BLT.05.029181 16628302 PMC2627322

[10] Spiegel PB. Who should be undertaking population-based surveys in humanitarian emergencies? Emerg Themes Epidemiol. 2007;4:12. doi: 10.1186/1742-7622-4-12 17543107 PMC1896153

[11] Carlin JB, Hocking J. Design of cross-sectional surveys using cluster sampling: an overview with Australian case studies. Aust N Z J Public Health. 1999;23:546–51. doi: 10.1111/j.1467-842X.1999.tb01317.x 10575783

[12] Stevenson MA. Sample size estimation in veterinary epidemiologic research. Front Vet Sci. 2020;7:539573. doi: 10.3389/fvets.2020.539573 33681313 PMC7925405

[13] Katz J, Carey VJ, Zeger SL, Sommer A. Estimation of design effects and diarrhea clustering within households and villages. Am J Epidemiol. 1993;138:994–1006. doi: 10.1093/oxfordjournals.aje.a116820 8256785

[14] Hermans V, Zachariah R, Woldeyohannes D, Saffa G, Kamara D, Ortuno-Gutierrez N, et al. Offering general pediatric care during the hard times of the 2014 Ebola outbreak: looking back at how many came and how well they fared at a Médecins Sans Frontières referral hospital in rural Sierra Leone. BMC Pediatr. 2017;17:34. doi: 10.1186/s12887-017-0786-z 28122533 PMC5264448

[15] MSF. Sierra Leone: MSF suspends emergency paediatric and maternal services in Gondama. 2014. Available from: https://www.msfindia.in/sierra-leone-msf-suspends-emergency-paediatric-and-maternal-services-gondama/

[16] Salama P, Assefa F, Talley L, Spiegel P, van Der Veen A, Gotway CA. Malnutrition, measles, mortality, and the humanitarian response during a famine in Ehiopia. JAMA. 2001;286:563–571. doi: 10.1001/jama.286.5.563 11476658

[17] Depoortere E, Checchi F, Broillet F, Gerstl S, Minetti A, Gayraud O, et al. Violence and mortality in West Darfur, Sudan (2003-04): epidemiological evidence from four surveys. Lancet. 2004;364:1315–1320. doi: 10.1016/S0140-6736(04)17187-0 15474133

[18] Burnham G, Lafta R, Doocy S, Roberts L. Mortality after the 2003 invasion of Iraq: a cross-sectional cluster sample survey. Lancet. 2006;368:1421–8. doi: 10.1016/S0140-6736(06)69491-9 17055943

[19] WHO. Sierra Leone: a traditional healer and a funeral. 2014. Available from: https://www.who.int/news/item/01-09-2015-sierra-leone-a-traditional-healer-and-a-funeral

[20] Martyn KK, Belli RF. Retrospective data collection using event history calendars. Nurs Res. 2002;51:270–274. doi: 10.1097/00006199-200207000-00008 12131240

[21] Caleo G, Duncombe J, Jephcott F, Lokuge K, Mills C, Looijen E, et al. The factors affecting household transmission dynamics and community compliance with Ebola control measures: a mixed-methods study in a rural village in Sierra Leone. BMC Public Health. 2018;18:248. doi: 10.1186/s12889-018-5158-6 29439682 PMC5812186

[22] MSF. Household-based survey of retrospective mortality rates, prevalence of malnutrition, and measles vaccination coverage (Districts of Bo and Pujehun, Sierra Leone). 2012.

[23] Bank W. Death rate, crude (per 1000 people) - Sierra Leone. 2014.

[24] Bilukha OO. Old and new cluster designs in emergency field surveys: in search of a one-fits-all solution. Emerg Themes Epidemiol. 2008;5:7. doi: 10.1186/1742-7622-5-7 18611276 PMC2474831

[25] WHO. Training for mid-level managers: the EPI coverage survey. Geneva: WHO Expanded Programme on Immunization; 1991. Available from: https://www.who.int/immunization/documents/MLM_module7.pdf

[26] WHO. Case definition recommendations for Ebola or Marburg virus diseases. 2014. Available from: https://apps.who.int/iris/bitstream/handle/10665/146397/WHO_EVD_CaseDef_14.1_eng.pdf

[27] Alpren C, Jalloh MF, Kaiser R, Diop M, Kargbo S, Castle E, et al. The 117 call alert system in Sierra Leone: from rapid Ebola notification to routine death reporting. BMJ Glob Health. 2017;2:e000392. doi: 10.1136/bmjgh-2017-000392 PMC5595198 28948044

[28] Miglietta A, Solimini A, Djeunang Dongho GB, Montesano C, Rezza G, Vullo V, et al. The Ebola virus disease outbreak in Tonkolili district, Sierra Leone: a retrospective analysis of the viral haemorrhagic fever surveillance system, July 2014–June 2015. Epidemiol Infect. 2019;147:e103. doi: 10.1017/S0950268819000177 30869055 PMC6518516

[29] Kolie D, Camara BS, Delamou A, Béavogui AH, Hermans V, Edwards JK, et al. The Ebola-effect in Guinea 2014-15: tangled trends of malaria care in children under-five. PLoS One. 2018;13:e0192798. doi: 10.1371/journal.pone.0192798 29489836 PMC5830300

[30] Lee-Kwan SH, DeLuca N, Bunnell R, Clayton HB, Turay AS, Mansaray Y. Facilitators and barriers to community acceptance of safe, dignified medical burials in the context of an Ebola epidemic, Sierra Leone, 2014. J Health Commun. 2017;22:24–30. doi: 10.1080/10810730.2016.1209601 28854130

[31] Alpren C, Jalloh MF, Kaiser R, Diop M, Kargbo S, Castle E, et al. The 117 call alert system in Sierra Leone: from rapid Ebola notification to routine death reporting. BMJ Global Health. 2017;2:e000392. doi: 10.1136/bmjgh-2017-000392 PMC5595198 28948044

[32] Development MoLGaR. Bye Laws for all chiefdoms in Sierra Leone. 2014. Available from: https://www.humanitarianresponse.info/sites/www.humanitarianresponse.info/files/documents/files/by-laws.pdf

[33] Caleo G, Theocharaki F, Lokuge K, Weiss HA, Inamdar L, Grandesso F, et al. Clinical and epidemiological performance of WHO Ebola case definitions: a systematic review and meta-analysis. Lancet Infect Dis. 2020;20:1324–38. doi: 10.1016/S1473-3099(20)30193-6 32593318 PMC9355392

[34] Vygen S, Tiffany A, Rull M, Ventura A, Wolz A, Jambai A, et al. Changes in health-seeking behavior did not result in increased all-cause mortality during the Ebola outbreak in Western Area, Sierra Leone. Am J Trop Med Hyg. 2016;95:897–901. doi: 10.4269/ajtmh.16-0295 27458039 PMC5062797

[35] Kuehne A, Lynch E, Marshall E, Tiffany A, Alley I, Bawo L, et al. Mortality, morbidity and health-seeking behaviour during the Ebola epidemic 2014–2015 in Monrovia results from a mobile phone survey. PLoS Negl Trop Dis. 2016;10:e0004899. doi: 10.1371/journal.pntd.0004899 27551750 PMC4994996

[36] Miller AC, Rohloff P, Blake A, Dhaenens E, Shaw L, Tuiz E, et al. Feasibility of satellite image and GIS sampling for population representative surveys: a case study from rural Guatemala. Int J Health Geogr. 2020;19:56. doi: 10.1186/s12942-020-00250-0 33278901 PMC7718677

[37] Kostoulas P, Nielsen SS, Browne WJ, Leontides L. Sample size estimation to substantiate freedom from disease for clustered binary data with a specific risk profile. Epidemiol Infect. 2013;141:1318–27. doi: 10.1017/S0950268812001938 22954371 PMC9151856

[38] Capture-recapture and multiple-record systems estimation II: applications in human diseases. International working group for disease monitoring and forecasting. Am J Epidemiol. 1995;142:1059–68. doi: 10.1093/oxfordjournals.aje.a117559 7485051

[39] Gignoux E, Idowu R, Bawo L, Hurum L, Sprecher A, Bastard M, et al. Use of capture-recapture to estimate underreporting of Ebola virus disease, Montserrado County, Liberia. Emerg Infect Dis. 2015;21:2265–2267. doi: 10.3201/eid2112.150756 26583831 PMC4672419

[40] Vallée J, Souris M, Fournet F, Bochaton A, Mobillion V, Peyronnie K, et al. Sampling in health geography: reconciling geographical objectives and probabilistic methods. An example of a health survey in Vientiane (Lao PDR). Emerg Themes Epidemiol. 2007;4:6. doi: 10.1186/1742-7622-4-6 17543100 PMC1894791

[41] Singh P, Pandey A, Aggarwal A. House-to-house survey vs. snowball technique for capturing maternal deaths in India: a search for a cost-effective method. Indian J Med Res. 2007;125:550–6. 17598941

[42] Sudman S, Freeman HE. The use of network sampling for locating the seriously ill. Med care. 1988;26:992–999. doi: 10.1097/00005650-198810000-00007 3172869

[43] Siddiqui NA, Rabidas VN, Sinha SK, Verma RB, Pandey K, Singh VP, et al. Snowball vs. House-to-house technique for measuring annual incidence of Kala-azar in the higher endemic blocks of Bihar, India: a comparison. PLoS Negl Trop Dis. 2016;10:e0004970. doi: 10.1371/journal.pntd.0004970 27681709 PMC5040448

[44] Hanage W, Qiu X, y Kennedy-Shaffer L. Snowball sampling study design for serosurveys in the early COVID-19 pandemic. Harvard Library Office for Scholarly Communication; 2020. Available from: http://bitlyws/9CVL.

[45] Roberts B, Morgan OW, Sultani MG, Nyasulu P, Rwebangila S, Myatt M, et al. A new method to estimate mortality in crisis-affected and resource-poor settings: validation study. Int J Epidemiol. 2010;39:1584–96. doi: 10.1093/ije/dyq188 21044978 PMC2992632

